# The association of health behavioral risk factors with quality of life in northern Sweden—A cross‐sectional survey

**DOI:** 10.1002/jgf2.333

**Published:** 2020-08-05

**Authors:** Ali K. Q. Al‐Rubaye, Klara Johansson, Laith Alrubaiy

**Affiliations:** ^1^ Basra Health Directorate Basra Iraq; ^2^ Department of Epidemiology and Global Health Umeå University Umeå Sweden; ^3^ St Mark’s Hospital London UK

**Keywords:** behavioural science, community medicine, health policy

## Abstract

**Background:**

It is well known that behavioral risk factors such as obesity, smoking, physical activity, diet, and excessive alcohol are linked to general health in northern Sweden. This study aimed to explore the joint relationship between these risk factors and the quality of life (QoL).

**Methods:**

Data were collected from Sweden's national public health survey between February and May 2014 in the four northern counties in Sweden. QoL was assessed using the EuroQol (EQ‐5D). Multivariable regression analysis was used to examine the relationship between five risk factors: BMI, physical activity, smoking status, fruit and vegetable intake, and alcohol consumption and QoL.

**Results:**

Data from 17 138 complete questionnaires showed that individuals who were not obese, did at least 30 minutes of physical activity daily, consumed at least 3 portions of vegetable or fruits, were not smoking daily, and who did not report being drunk at least once every week were found to have better QoL (*P* < .005). The mean EQ‐5D score ranged from 0.85 to 0.79. Approximately, two thirds of the studied population reported being physically active for at least 30 minutes every day and two fifths of them had a normal BMI. Only around 7% of the sample reported that they were eating the recommended daily level of fruits and vegetables.

**Conclusions:**

The results of the study suggest that QoL has a significant relationship with lifestyle behaviors. This finding would emphasize the role of interventions to improve population health.

## Graphical abstract

Data from 17 138 complete questionnaires showed that individuals who were not obese, did at least 30 minutes of physical activity daily, consumed at least 3 portions of vegetable or fruits, were not smoking daily, and who did not report being drunk at least once every week were found to have better quality of life (*P* < .005).

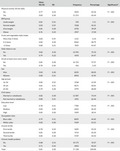

## 1 INTRODUCTION

Obesity, smoking, low level of physical activity, lack of fruit and vegetable intake, and harmful consumption of alcohol, all are established risk factors that have undesirable effects on health. These behavioral risk factors are potentially preventable to avoid their adverse effect on health^1^, ^2^, ^3^, ^4^ as well as their potential economic consequences. Modifying these risk factors is important to be considered in interventions to improve public health.

Overweight and obese individuals have been highly stigmatized and discriminated, and they are seen as lazy, less motivated, and unattractive; therefore, they often experience social discrimination in different settings^5^. The negative stereotype stigma of obese individuals in the society may increase the feel of shame and affect their psychological health, and they are more likely to lead to poor QoL^5^, ^6^. Higher levels of physical activity have been shown to be associated with better QoL^7^, ^8^. Snus is a smokeless tobacco product commonly used by Swedes. It is a moist tobacco powder originating from a variant of dry snuff^9^, ^10^. There is an inverse relationship between the amount of tobacco and the QoL. Light smokers have a better level of QoL than the heavy smokers^11^. Many studies have shown that smokers in general express lower quality of life compared with the nonsmokers^11^, ^12^, ^13^, ^14^, ^15^. They tend to report sleep disorders, symptoms of anxiety, and depression more than the nonsmokers, and they are more likely to have unhealthy diet style and to be physically inactive^11^. In addition to health benefits, regular consumption of fruit and vegetable improves QoL and reduces the risk of chronic illnesses^16^, ^17^.

The recommended upper limit for alcohol intake in Sweden is up to 14 drinks per week and there should be no more than 5 drinks unit at once for men, while the recommended upper limit for alcohol intake for women is up to 9 drinks per week with no more than 4 at one occasion^18^. Heavy episodic drinking, or binge drinking, is a term used for the overconsumption of alcohol with an intention of becoming intoxicated, and this typically happens when a person's blood alcohol concentration reaches the level of 0.08 grams percent or above. It is found that the habit of binge drinking is significantly associated with worse QoL, and it leads to many serious health problems ranging from unintentional injuries and violence including homicide and suicide to the developing of many chronic diseases such as high blood pressure, stroke, and heart disease, and the developing of cancers in different parts in the body^4^, ^18^, ^19^.

This study will aim to provide a population‐based evidence on the joint association of obesity, physical activity, tobacco use, low consumption of fruit and vegetable, and binge drinking together on the QoL in a population sample of adults from northern Sweden. In so doing, these findings may provide a significant new resource to inform the cost‐effectiveness of interventions aimed at tackling these major public health concerns in the studied population.

## 2 METHODS

### 2.1 Study population

This cross‐sectional study is based on data from Sweden's national public health survey Hälsa på lika villkor—HLV (Health on Equal Terms)—that was conducted between February and May 2014 in the four northern counties in Sweden: Norrbotten, Västerbotten, Västernorrland, and Jämtland. The HLV survey is a series of public surveys, the extended version of the HLA survey of the northern Sweden which is conducted every 4 years. The survey has been administrated by the Public Health Agency of Sweden (Swedish: Folkhälsomyndigheten), in collaboration with the county councils and Statistics Sweden. The questionnaire consisted of 18 pages with a total of 85 questions. The use of the “Health on Equal Terms” survey in the present study was reviewed and approved by the ethical committee at the Regional Ethical Review Board in Umeå (2015/134‐31Ö).

Many of the survey questions originated from a “Survey of Living Conditions” that was done in Stockholm, Skåne, and northern counties and verified in a pilot study in 2003. The survey questions cover physical and mental health, consumption of pharmaceuticals, contact with healthcare services, dental health, living habits, financial conditions, work and occupation, work environment, safety, and social relationships. We followed the STROBE statement guidance in reporting the HLV survey results^20^.

A stratified random sampling (by age, sex, and municipality) was used in the survey, among all people with the age between 16 and 84 years who were residents in the four northmost counties of Sweden (Västerbotten, Norrbotten, Västernorrland, and Jämtland). The questionnaires were sent out by regular mail, and the participants were asked to answer the questions and send their answers back to Statistics Sweden. The respondents were informed that the survey was voluntary; it was also possible to answer on the Internet. They received login information in the form of a user name and a password and could then log in via Statistics Sweden's website. On the Web, there was also the possibility to answer the questions in English or Finnish. Checking that the right person has answered the questionnaire has been done by comparing answers to questions about birth year and gender with the corresponding record.

### 2.2 Quality of Life (QoL)

EuroQol (EQ‐5D) was used to measure QoL^21^, ^22^, ^23^. It provides a simple and generic measure of health, and it provides a descriptive profile and a single numeric value for the state of the individual health that can be used in different settings. It contains two parts, one is the descriptive system and the other is the visual analogue scale. The respondent is asked to self‐rate his current health state as well as mobility, self‐care, usual activities, pain/discomfort, and anxiety/depression.

### 2.3 Health behavioral risk factors

#### 2.3.1 Body mass index (BMI)

The respondents self‐reported their height and weight, and they were categorized in four groups according to their BMI: underweight, when the BMI <18.5 kg/m^2^; normal weight, when BMI is between 18.5 and 25 kg/m^2^; overweight, when it is between 25 and 30 kg/m^2^; or obese if the value is over 30 kg/m^2^ ^24^. The normal weight group was selected as the reference group in the analysis.

#### 2.3.2 Alcohol consumption

The participants were asked in the survey “How often during the past 12 months have you drunk so much alcohol that you have become drunk?” and the answers for that question were used to categorize the participants into two groups, the first group represent those who were consuming alcohol to the extent that they were feeling drunk at least once every week, and the other group involved the participants who report being drunk less frequently. These two groups were used in the study to analyze the association of binge drinking on the EQ‐5D.

#### 2.3.3 Smoking habits

The participants were asked whether they were smoking and/or using snus daily or not, and the participants were grouped into two groups, the first group was those who replied that they were not smoking and were not using snus daily, and the other group was those who replied that they were smoking and/or using snus daily.

#### 2.3.4 Fruit and vegetable consumption

The participants were divided into three groups according to their total daily fruit and vegetable consumption: The first group was those who were eating <2 portions per day, the second group was those who were eating 2‐4 portions per day, and the last group was those who were eating a total of more than 4 portions per day. The last group was used as the reference group in the analysis.

#### 2.3.5 Sociodemographic characteristics

Sex, age, marital/civil status, educational level, occupation and income, and long‐term health problems were collected for the study participants to rule out potential confounders in multivariable analyses.

For education, the participants were divided into three groups depending on their education level. The first group was the low education‐level group which involved the participants who complete <3 years in upper‐secondary education, and the second group was the medium education‐level group and it involved the participants who complete at least 3 years in upper‐secondary education but <3 years in postsecondary education. The final group was the high education‐level group which involved those who complete at least 3 years in postsecondary education. The education‐level variable was treated as a categorical variable, and the participants with high education level were used as the reference group in the analysis.

For occupation, the participants were divided into two groups depending on the type of their occupation which was retrieved from register data, the blue‐collar worker group and white‐collar worker group. The blue‐collar worker consists of both the unskilled and the skilled manual workers, while the white collar consists of assistant nonmanual, intermediate nonmanual, professionals, and self‐employed. The white‐collar group was used as the reference group in the analysis.

For income, the participants were divided into three groups depending on their personal disposable income level, the first group included participants with the lowest third of income, the second group included participants with the middle third of income, and the last group included participants with the highest third of income. Income was treated as a categorical variable, and the group that included participants with the highest third of income was used as the reference group. The data of income were collected from the income and taxation register.

### 2.4 Statistical analysis

The statistical analyses were performed with Stata/MP 13.0 software. Multivariable linear regression was used to investigate the relationship between the EQ‐5D utility score (dependent variable) and health behavioral risk factors (BMI, alcohol consumption, smoking status, fruit and vegetable intake, and physical activity) and the potential confounders (age, sex, civil status, education level, occupation class, income, and the presence of any chronic health problem). Sensitivity analyses were done by removing the variables with high and biased dropout and repeating the multivariable linear regression.

Finally, three linear regression models on the EQ‐5D utility score were performed. Model 1 shows the results of the regression of behavioral lifestyle factors adjusted to each other on the EQ‐5D, Model 2 is the regression further adjusted for sex, age, education level, and civil status, while the regression in Model 3 is further adjusted for occupation classes, income, and chronic illnesses.

## 3 RESULTS

With a response rate of 50%, we had 25 667 returned questionnaires. Questionnaires with missing data were dropped to ensure complete analysis. We analyzed 17 138 (67%) completed questionnaires. Binge drinking and the occupation were the most missed items. Sensitivity analysis by removing binge drinking and occupation variables and repeating the multiple regression analysis did not show a significant difference in results.

Approximately, two thirds of the individuals reported being physically active for at least 30 minutes every day and two fifths of them had a normal BMI (Table 1). Only around 7% of the sample reported that they were eating the recommended daily level of fruits and vegetables. Regarding the tobacco use and alcohol consumption, only around 3% of the sample reported being drunk at least once every week and about one quarter of them were using a daily tobacco.

**Table 1:** Descriptive statistics for the EQ‐5D index score across health behavioral risk factors and socioeconomic characteristics (n = 17 138)

	Mean EQ‐5D	SD	Frequency	Percentage	Significance^a^
Physical activity 30 min daily					
No	0.77	0.23	5923	34.56	*P* < .001
Yes	0.83	0.18	11 215	65.44	
BMI group					
Underweight	0.81	0.21	190	1.11	*P* < .001
Normal weight	0.83	0.19	7268	42.41	
Overweight	0.81	0.20	6733	39.29	
Obese	0.76	0.23	2947	17.20	
Fruits and vegetables daily intake					
More than 4 times	0.83	0.19	1121	6.54	*P* < .001
2‐4 times	0.82	0.20	8362	48.79	
<2 times	0.80	0.21	7655	44.67	
Daily tobacco use					
No	0.82	0.20	12 945	75.53	*P* < .001
Yes	0.79	0.22	4193	24.47	
Drunk at least once every week					
No	0.81	0.20	16 722	97.57	*P* < .001
Yes	0.78	0.24	416	2.43	
Sex					
Men	0.83	0.20	8235	48.05	*P* < .001
Women	0.80	0.21	8903	51.95	
Age groups					
16‐34	0.85	0.19	3768	21.99	*P* < .001
35‐64	0.81	0.21	8571	50.01	
65‐84	0.79	0.20	4799	28.00	
Civil status					
Married or cohabitants	0.82	0.20	12 587	73.44	*P* < .001
Not married or cohabitants	0.80	0.21	4551	26.56	
Education level					
Low	0.78	0.21	7785	45.43	*P* < .001
Medium	0.83	0.20	6244	36.43	
High	0.85	0.18	3109	18.14	
Occupation class					
Blue collar	0.79	0.21	8029	46.85	*P* < .001
White collar	0.83	0.20	9109	53.15	
Income tertile					
First tertile	0.78	0.22	5695	33.23	*P* < .001
Second tertile	0.81	0.20	5714	33.34	
Third tertile	0.85	0.18	5729	33.43	
Chronic health problem					
No	0.88	0.13	10 175	59.37	*P* < .001
Yes	0.71	0.24	6963	40.63	
Total	0.81	0.20	17 138	100.00	

^a^ The *P* values were calculated using the *t* test for the binary variables and one‐way ANOVA for the variables with multiple categories.

Women tended to be more in normal weight group, while men tended to be more in the overweight group (Table 2). With regard to physical activity, there was no significant difference between men and women. While more than half of the men tended to eat less than two portions of fruits and vegetables daily, more than half of the women tended to eat between 2 and 4 portions from fruits or vegetables daily. Regarding the daily tobacco use, women reported daily tobacco use more than men however men reported being drunk at least once weekly more than women.

**Table 2:** Distribution of the study sample across health behavioral risk factors and socioeconomic characteristics stratified by sex (n = 17 318)

Variables	Men		Women		chi‐2
	Freq	%	Freq	%	
BMI groups					
Underweight	39	0.47%	151	1.70%	*P* < .001
Normal weight	2875	34.91%	4393	49.34%	
Overweight	3863	46.91%	2870	32.24%	
Obese	1458	17.70%	1489	16.72%	
30 min of daily physical activity					
Yes	5386	65.40%	5829	65.47%	*P* = .925
No	2849	34.60%	3074	34.53%	
Daily tobacco use					
No	5607	68.09%	7338	82.42%	*P* < .001
Yes	2628	31.91%	1565	17.58%	
Daily fruit and vegetable intake					
More than 4 times	269	3.27%	852	9.57%	*P* < .001
2‐4 times	3238	39.32%	5124	57.55%	
<2 times	4728	54.71%	2927	32.88%	
Being drunk once weekly					
No	7910	96.05%	8812	98.98%	*P* < .001
Yes	325	3.95%	91	1.02%	
Age groups					
16‐34	1603	19.47%	2165	24.32%	*P* < .001
35‐64	4059	49.29%	4512	50.68%	
64‐85	2573	31.24%	2226	25.00%	
Civil status					
Married	5999	72.85%	6588	74.00%	*P* = .089
Not married	2236	27.15%	2315	26.00%	
Occupation class					
White collar	4130	50.15%	4979	55.92%	*P* < .001
Blue collar	4105	49.85%	3924	44.08%	
Education level					
Low	4073	49.46%	3712	41.69%	*P* < .001
Medium	3096	37.60%	3148	35.36%	
High	1066	12.94%	2043	22.95%	
Income level					
1st tertile	2211	26.85%	3484	39.13%	*P* < .001
2nd tertile	2345	28.48%	3369	37.84%	
3rd tertile	3679	44.68%	2050	23.03%	
Chronic health problem					
No	4787	58.13%	5388	60.52%	*P* < .001
Yes	3448	41.87%	3515	39.48%	
Total	8235	48.05%	8903	51.95%	17.138

The mean EQ‐5D utility index score for the total study population was 0.811 with standard deviation of 0.204. Respondents who reported being active at least 30 minutes every day, in normal BMI group, eating the recommended daily level of fruits and vegetables, not smoking or using snus daily, and not report being drunk at least once every week had a significantly higher mean EQ‐5D compared with the other group in each category (*P* < .001).

Comparing the mean EQ‐5D across the socioeconomic and sociodemographic variables, it was found to be significantly higher in individuals who were men, those who were within the age group 16‐30 years, those who were married or cohabitating, had higher education, had higher income level, had a white‐collar occupation, and did not have any long‐term health (*P* < .001).

Table 3 shows the results of the multivariable linear regression with EQ‐5D regressed on behavioral lifestyle risk factors, while holding different sociodemographic and socioeconomic characteristics constant. Model 1 shows the results of the regression of behavioral lifestyle factors adjusted to each other on the EQ‐5D. Model 2 is the regression further adjusted for sex, age, education level, and civil status. Model 3 is further adjusted for occupation classes, income, and chronic illnesses. The R2 in Model 1 was 0.04, and it was increasing with subsequent models as the regression further adjusted to the socioeconomic and sociodemographic characteristics.

**Table 3:** Linear regression with EQ‐5D regressed on lifestyle risk factors and sociodemographic and socioeconomic characteristics (n = 17 138)

	Model 1		Model 2		Model 3	
	Coef.	[CI 95%]	Coef.	[CI 95%]	Coef.	[CI 95%]
Physically active 30 min/d	0.056 ***	[0.049, 0.062]	0.050***	[0.044, 0.057]	0.037***	[0.031, 0.043]
BMI groups						
Underweight	−0.018	[−0.046, 0.011]	−0.019	[−0.048, 0.009]	−0.007	[−0.033, 0.019]
Normal weight	0	0	0	0	0	0
Overweight	−0.014***	[−0.021, −0.007]	−0.014***	[−0.021, −0.007]	−0.007*	[−0.013, −0.001]
Obese	−0.058***	[−0.067, −0.049]	−0.056***	[−0.064, −0.047]	−0.035***	[−0.043, −0.027]
Being drunk at least once every week	−0.017	[−0.036, 0.003]	−0.032**	[−0.052, −0.013]	−0.025**	[−0.042, −0.007]
Smoking/snus daily	−0.024***	[−0.031, −0.017]	−0.033***	[−0.040, −0.026]	−0.025***	[−0.031, −0.018]
Number of times eating vegetables or fruits every day						
More than 4	0	0	0	0	0	0
2‐4	−0.002	[−0.015, 0.010]	−0.004	[−0.016, 0.009]	−0.005	[−0.016, 0.006]
<2	−0.008	[−0.020, 0.005]	−0.022***	[−0.035, −0.009]	−0.016**	[−0.027, −0.004]
Age groups						
16‐34 y			0	0	0	0
35‐64 y			−0.039***	[−0.047, −0.031]	−0.034***	[−0.042, −0.026]
65‐84 y			−0.067***	[−0.076, −0.058]	−0.029***	[−0.037, −0.020]
Sex						
Men			0	0	0	0
Women			‐0.045***	[−0.051, −0.039]	−0.037***	[−0.043, −0.031]
Civil status						
Married/cohabitants			0	0	0	0
Not married/not cohabitants			−0.019***	[−0.026, −0.012]	−0.004	[−0.010, 0.002]
Education level						
Low					0	0
Medium					0.015***	[0.009, 0.022]
High					0.022***	[0.013, 0.030]
Occupation						
Blue collar/Manu. worker					0	0
White collar/non‐Manu. worker					0.011***	[0.005, 0.018]
Income						
First tertile					0	0
Second tertile					0.018***	[0.011, 0.025]
Third tertile					0.044***	[0.036, 0.052]
Chronic illness						
No					0	0
Yes					−0.162***	[−0.168, −0.157]
Constant	0.802***	[0.789, 0.815]	0.881***	[0.866, 0.897]	0.887***	[0.872, 0.903]
R‐sqr	0.04		0.06		0.22	

Note

Model 1: unadjusted regression of the lifestyle risk factors with the EQ‐5D.

Model 2: the regression of the covariates with the EQ‐5D adjusted to the sex, age, education level, and civil status.

Model 3: Model 2 plus adjusting to the occupation class, income, and chronic illnesses.

* *P*‐value < .05.

** *P*‐value < .01.

*** *P*‐value < .001.

The association of daily physical activity and tobacco use with the EQ‐5D was significant (*P* < .001) across all the three models (Table 3). Being drunk at least once every week was correlated with EQ‐5D in Model 2 and Model 3 (*P* < .05). Obesity was strongly correlated with EQ‐5D across all the three models (*P* < .001). However, there was not a significant difference in the EQ‐5D scores in the underweight and overweight groups. Consumption of fruits or vegetables daily was significantly associated with EQ‐5D in Model 2 and Model 3.

When linear regression was stratified by sex (Tables 4 and 5), the daily tobacco use, low level of daily physical activity, and being obese remain associated with low EQ‐5D (*P* < .001). Being overweight was significantly associated with low EQ‐5D score in women in Model 1 and Model 2, but not in Model 3, while in men overweight was significantly associated with low EQ‐5D only in Model 1. Fruit and vegetable consumption was not found to have any association with the EQ‐5D in men.

**Table 4:** Linear regression with EQ‐5D regressed on lifestyle risk factors and sociodemographic and socioeconomic characteristics stratified by sex: male (n = 8235)

Regression stratified by sex: male	Model 1		Model 2		Model 3	
	Coef.	[CI 95%]	Coef.	[CI 95%]	Coef.	[CI 95%]
Physically active 30 min/d	0.053 ***	[0.044, 0.062]	0.050***	[0.041, 0.059]	0.038***	[0.029, 0.046]
BMI groups						
Underweight	−0.042	[−0.103, 0.020]	−0.052	[−0.113, 0.009]	−0.034	[−0.090, 0.022]
Normal weight	0	0	0	0	0	0
	−0.014**	[−0.024, −0.005]	−0.008	[−0.018, 0.001]	−0.007	[−0.015,0.002]
Obese	−0.046***	[−0.058, −0.034]	−0.040***	[−0.053, −0.028]	−0.027***	[−0.038, −0.015]
Being drunk at least once every week	−0.028*	[−0.049, −0.006]	−0.036**	[−0.058, −0.015]	−0.025**	[−0.045, −0.006]
Smoking/snus daily	−0.021***	[−0.03, −0.012]	−0.025***	[−0.034, −0.016]	−0.018***	[−0.026, −0.009]
Number of times eating vegetables or fruits every day						
More than 4	0	0	0	0	0	0
2‐4	−0.014	[−0.038, 0.011]	−0.010	[−0.034, 0.014]	−0.006	[−0.027, 0.016]
<2	−0.019	[−0.043, 0.005]	−0.019	[−0.043, 0.005]	−0.011	[−0.033, 0.011]
Age groups						
16‐34 y			0	0	0	0
35‐64 y			−0.048***	[−0.060, −0.036]	−0.037***	[−0.048, −0.025]
65‐84 y			−0.089***	[−0.101, −0.076]	−0.041***	[−0.053, −0.028]
Civil status						
Married/cohabitants			0	0	0	0
Not married/not cohabitants			−0.019***	[−0.029, −0.09]	−0.000	[−0.009, 0.009]
Education level						
Low					0	0
Medium					0.021***	[0.0012, 0.030]
High					0.031***	[0.018, 0.040]
Occupation						
Blue collar/Manu. worker					0	0
White collar/non‐Manu. worker					0.015***	[0.007, 0.024]
Income						
First tertile					0	0
Second tertile					0.011*	[0.000, 0.021]
Third tertile					0.044***	[0.034, 0.055]
Chronic illness						
No					0	0
Yes					−0.151***	[−0.159, −0.143]
Constant	0.805***	[0.805, 0.856]	0.886***	[0.859, 0.913]	0.887***	[0.850, 0.903]
R‐sqr	0.03		0.06		0.22	

Note

Model 1: unadjusted regression of the lifestyle risk factors with the EQ‐5D.

Model 2: the regression of the covariates with the EQ‐5D adjusted to the sex, age, education level, and civil status.

Model 3: Model 2 plus adjusting to the occupation class, income, and chronic illnesses.

* *P*‐value < .05.

** *P*‐value < .01.

*** *P*‐value < .001.

**Table 5:** Linear regression with EQ‐5D regressed on lifestyle risk factors and sociodemographic and socioeconomic characteristics stratified by sex: female (n = 8903)

Regression stratified by sex: female	Model 1		Model 2		Model 3	
	Coef.	[CI 95%]	Coef.	[CI 95%]	Coef.	[CI 95%]
Physically active 30 min/d	0.053 ***	[0.044, 0.062]	0.051***	[0.042, 0.06]	0.036***	[0.028, 0.044]
BMI groups						
Underweight	−0.02	[−0.035, 0.031]	−0.009	[−0.042, 0.024]	−0.028	[−0.028, 0.032]
Normal weight	0	0	0	0	0	0
Overweight	−0.026***	[−0.035, −0.016]	−0.020***	[−0.029, −0.01]	−0.005	[−0.014, 0.004]
Obese	−0.075***	[−0.087, −0.063]	−0.070***	[−0.082, −0.058]	−0.042***	[−0.053, −0.031]
Being drunk at least once every week	−0.025	[−0.067, 0.017]	−0.03	[−0.072, 0.012]	−0.032	[−0.070, 0.007]
Smoking/snus daily	−0.044***	[−0.055, −0.033]	−0.045***	[−0.056, −0.033]	−0.035***	[−0.045, −0.025]
Number of times eating vegetables or fruits every day						
More than 4	0	0	0	0	0	0
2‐4	−0.003	[−0.018, 0.012]	−0.000	[−0.015, 0.015]	−0.004	[−0.018, 0.009]
<2	−0.026**	[−0.041, −0.010]	−0.026***	[−0.042, −0.011]	−0.019**	[−0.033, −0.005]
Age groups						
16‐34 y			0	0	0	0
35‐64 y			−0.035***	[−0.046, −0.024]	−0.034***	[−0.045, −0.023]
65‐84 y			−0.046***	[−0.059, −0.034]	−0.017**	[−0.029, −0.005]
Civil status						
Married/cohabitants			0	0	0	0
Not married/not cohabitants			−0.022***	[−0.032, −0.012]	−0.009*	[−0.018, 0.000]
Education level						
Low					0	0
Medium					0.010*	[0.001, 0.020]
High					0.016**	[0.004, 0.028]
Occupation						
Blue collar/Manu. worker					0	0
White collar/non‐Manu. worker					0.008	[−0.001, 0.017]
Income						
First tertile					0	0
Second tertile					0.026***	[0.016, 0.035]
Third tertile					0.042***	[0.030, 0.054]
Chronic illness						
No					0	0
Yes					−0.171***	[−0.179, −0.163]
Constant	0.803***	[0.786, 0.819]	0.835***	[0.817, 0.853]	0.859***	[0.840, 0.877]
R‐sqr	0.05		0.06		0.23	

Note

Model 1: unadjusted regression of the lifestyle risk factors with the EQ‐5D.

Model 2: the regression of the covariates with the EQ‐5D adjusted to the sex, age, education level, and civil status.

Model 3: Model 2 plus adjusting to the occupation class, income, and chronic illnesses.

* *P*‐value < .05.

** *P*‐value < .01.

*** *P*‐value < .001.

## 4 DISCUSSION

This study analyzed the association between the QoL measured by EQ‐5D‐3L score and the following five lifestyle risk factors: BMI, physical activity, fruit and vegetable intake, tobacco use, and binge drinking, in 16‐ to 85‐year‐old individuals from northern Sweden. The study results showed that there was a significant association between the EQ‐5D score and the study variables (BMI groups, 30 minutes of daily physical activity, daily fruit and vegetable consumption, daily tobacco use, and at least being drunk once a week). The study found that individuals who were not obese, did at least 30 minutes of physical activity daily, consumed at least 3 portions of vegetable or fruits, were not smoking or using snus daily, and who did not report being drunk at least once every week were found to report better EQ‐5D.

The mean EQ‐5D utility index score for the study population ranged from 0.85 (in 16‐34 years) to 0.79 (in 65‐84 years). The results support previous findings in a study that describes the EQ‐5D index values in the general population in Stockholm County which ranged from 0.89 (in 20‐29 years) to 0.74 (80‐88 years)^25^.

The study showed that among lifestyle behavioral factors, obesity and low physical activity had the strongest association with low QoL in the participants. The inverse relationship between these two lifestyle factors and the QoL found in this study were consistent with the findings found in previous studies from different parts of the world such as in China, Spain, and the United States^26^, ^27^, ^28^.

The study found that the association between overweight and low EQ‐5D is less than what it was with obesity, and it seemed to be confounded by other variables. These findings were similar to what was found in a study previously done in the general population in the UK where it showed that underweight was not associated with a significant reduction in EQ‐5D score and the overweight association with low EQ‐5D was becoming nonsignificant when the regression was adjusted to other sociodemographic factors^3^.

As expected, daily tobacco use and the heavy episodic drinking of alcohol were found to be associated with a significant decrease in the QoL. These results are supporting findings in a previous study that tobacco use and heavy alcohol drinking associated with significant reduction in QoL^3^, ^4^, ^11^, ^18^. We opted to use a more rare but severe outcome of a harmful pattern of alcohol use rather than a general measure of total alcohol consumption, since we assessed this to be the most relevant outcome in relation to QoL. The study showed that 2.43% of the surveyed population reported being drunk at least once every week. Further studies are, of course, needed to clarify this correlation.

The study showed that while low daily fruit and vegetable consumption was related to low QoL, it had the lower association compared with the other lifestyle characteristics. This finding was comparable to other studies^3^, ^11^, ^17^, ^29^. Therefore, it is possible that the benefits of “5‐a‐day” could well be closely related to these other health behaviors.

In the Swedish context, a study has shown that increasing weight above the normal range had a negative effect on the QoL even after the control of the major health risk factors and the sociodemographic characteristics^30^. Another study also conducted in Sweden suggests that obesity, not consuming adequate vegetables, and smoking among young people have independent associations with QoL^8^.

It was noteworthy to find that approximately one in three participants were inactive and that this had a similar independent association with lower EQ‐5D than being obese. Given that low levels of physical activity and high BMI, a common finding in national surveys, interventions to promote physical activity, and weight management are urgently needed. A minimally important difference in EQ‐5D utility score has previously been estimated at 0.0744; findings of reductions in EQ‐5D utility scores of greater than this for obesity and physical inactivity suggest that the estimated differences are clinically important.

The study also found that there are some differences between men and women (Tables 4 and 5). Obesity had a more significant association with low EQ‐5D in women compared with men (*P* < .001). This could be explained that weight perception and impact on the QoL are different between men and women^31^.

Although this survey provided a detailed assessment of health behavioral risk factors, apart from BMI, the measures were self‐reported and therefore open to biases as a consequence of potential under‐ or overreporting. The response rate to the survey was around 50%, and we excluded 8529 (33%) individuals because of incomplete data. However, sensitivity analysis by removing the most dropped variables and repeating the multiple regression analysis did not show a significant difference in results. The cross‐sectional design of the study does not assess the temporal relationship between exposure and outcome. The recall period for most of the survey was 12 months made the recall bias a possible limitation to the study.

The study used registration‐linked data to the Swedish personal number that allowed accurate matching of socioeconomic and sociodemographic characteristics. The consistency between our findings and approach to those of comparable studies adds credibility to our study^3^, ^11^, ^14^, ^31^.

The strength of this study was its use of a large cross‐sectional public survey. Although the study is based on the 2014 HLY survey, it represented a good representation of the northern Sweden population. Future studies would be recommended to analyze subsequent HLV surveys.

In summary, the results of the study show that QoL measured by EQ‐5D‐3L has a significant correlation with lifestyle behaviors. The findings suggest that behavioral risk factors namely obesity, less than the recommended daily level of physical activity, low daily consumption of fruits and vegetables, daily use of tobacco, and the heavy episodic drinking of alcohol may have a negative association with QoL. Our study highlights the importance of tackling these behavioral risk factors. It may serve as a ground to formulate new policies that aim to improve population QoL. Further research is needed to examine the longitudinal effect of public health measures on population QoL and overall health.

## **CONFLICT** OF **INTERESTS**

None.

## AUTHOR CONTRIBUTIONS

All authors contributed to research design, interpreted the data, and reviewed the drafts of the paper. AA performed the statistical analyses.

## ETHICAL APPROVAL

The use of the “Health on Equal Terms” survey in the present study was reviewed and approved by the ethical committee at the Regional Ethical Review Board in Umeå (2015/134‐31Ö). The participants had received information letters about the survey. Individuals’ identifying details have been removed for confidentiality. The necessary informed consents were obtained for the study when required.
